# Increased Dentate Gyrus Excitability in the Intrahippocampal Kainic Acid Mouse Model for Temporal Lobe Epilepsy

**DOI:** 10.3390/ijms25010660

**Published:** 2024-01-04

**Authors:** Marijke Vergaelen, Simona Manzella, Kristl Vonck, Erine Craey, Jeroen Spanoghe, Mathieu Sprengers, Evelien Carrette, Wytse Jan Wadman, Jean Delbeke, Paul Boon, Lars Emil Larsen, Robrecht Raedt

**Affiliations:** 1 4BRAIN, Department of Head and Skin, Ghent University, 9000 Ghent, Belgium; 2 MEDISIP, Department of Electronics and Information Systems, Ghent University, 9000 Ghent, Belgium

**Keywords:** temporal lobe epilepsy (TLE), intrahippocampal kainic acid (IHKA) mouse model, multielectrode array (MEA), field postsynaptic potential (fPSP), dentate gyrus (DG)

## Abstract

The intrahippocampal kainic acid (IHKA) mouse model is an extensively used in vivo model to investigate the pathophysiology of mesial temporal lobe epilepsy (mTLE) and to develop novel therapies for drug-resistant epilepsy. It is characterized by profound hippocampal sclerosis and spontaneously occurring seizures with a major role for the injected damaged hippocampus, but little is known about the excitability of specific subregions. The purpose of this study was to electrophysiologically characterize the excitability of hippocampal subregions in the chronic phase of the induced epilepsy in the IHKA mouse model. We recorded field postsynaptic potentials (fPSPs) after electrical stimulation in the CA1 region and in the dentate gyrus (DG) of hippocampal slices of IHKA and healthy mice using a multielectrode array (MEA). In the DG, a significantly steeper fPSP slope was found, reflecting higher synaptic strength. Population spikes were more prevalent with a larger spatial distribution in the IHKA group, reflecting a higher degree of granule cell output. Only minor differences were found in the CA1 region. These results point to increased neuronal excitability in the DG but not in the CA1 region of the hippocampus of IHKA mice. This method, in which the excitability of hippocampal slices from IHKA mice is investigated using a MEA, can now be further explored as a potential new model to screen for new interventions that can restore DG function and potentially lead to novel therapies for mTLE.

## 1. Introduction

The intrahippocampal kainic acid (IHKA) mouse model is an extensively used in vivo model to investigate mesial temporal lobe epilepsy (mTLE), the most common type of drug-resistant epilepsy (DRE). The model involves critical pathological features of human mTLE, such as the occurrence of drug-resistant spontaneous seizures and hippocampal sclerosis [1,2,3]. Therefore, this model is considered appropriate to study the pathophysiology of mTLE, and it has been used in the development of several FDA- and EMA-approved antiepileptic drugs (AEDs), including topirimate, carbamazepine, lamotrigine, and pregabalin [4,5,6,7].

Epilepsy is induced through the unilateral injection of the glutamate receptor agonist kainic acid (KA) in the dorsal hippocampus. Following KA injection, three different time frames can be identified in the intracerebral electroencephalography (EEG) recordings. Immediately after KA injection, the EEG is characterized by continuous seizure activity (status epilepticus, SE) that lasts for several hours, then a latent phase of 14–21 days with sporadic low voltage spike or spike-and-wave activity, followed by the chronic epilepsy phase, with spontaneous recurrent seizures originating from the hippocampus, which is then affected by sclerosis [3,8,9].

Hippocampal sclerosis after KA injection features morphological changes similar to hippocampal sclerosis in patients. It is characterized by a loss of neurons in the CA1, CA3, and hilar hippocampal regions, in combination with pronounced gliosis with reactive astrocytes and microglia. In the dentate gyrus (DG), there is a severe loss of inhibitory interneurons, while the excitatory granule cells remain spared. Moreover, the granule cell layer shows a striking structural reorganization, including dispersion and formation of new axonal connections with other granule cells, a process termed mossy fiber sprouting [3,8,9,10,11,12,13].

The pathophysiological changes in the DG of both mTLE patients and IHKA mice are believed to disrupt the so-called dentate gate function. In healthy conditions, granule cells display a low excitability which in combination with extensive feedforward and feedback inhibition turn the DG into an input filter towards the hippocampus. A disturbed dentate gate function results in excessive excitation emerging from or passing through the DG towards downstream regions, eventually causing seizures [11,14,15,16]. In this regard, the DG is potentially a more interesting target than the hippocampus as a whole for the development of therapies [11].

Although the IHKA model has been widely adopted for in vivo studies, it has rarely been investigated for ex vivo therapy testing purposes, and little is known about circuit excitability in hippocampal subregions [3,13,17,18]. The identification of subregion specific changes in circuit excitability could help focus therapy developments towards specific subregions, as made possible by upcoming therapies with high spatial resolution, such as gene therapy (chemo- and optogenetics) or photopharmacology [19,20].

A commonly used technique to evaluate hippocampal excitability in vivo or ex vivo consists of recording field postsynaptic potentials (fPSPs) evoked by electrical stimulation. The hippocampus is extremely suitable for these recordings through its highly structured anatomical organization with clearly identifiable in- and output layers. With spontaneous local field potentials, it is far more challenging to localize the origin and infer the state of excitability. In addition, for ex vivo hippocampal slices, the level of spontaneous activity is extremely low in physiological artificial cerebrospinal fluid (aCSF). The level of spontaneous activity can be increased by changing the ionic composition of aCSF and/or adding proconvulsive substances, but these alterations could interfere with the working mechanisms of potential therapies to be tested using ex vivo hippocampal slices [21,22,23,24].

**Figure 1:**
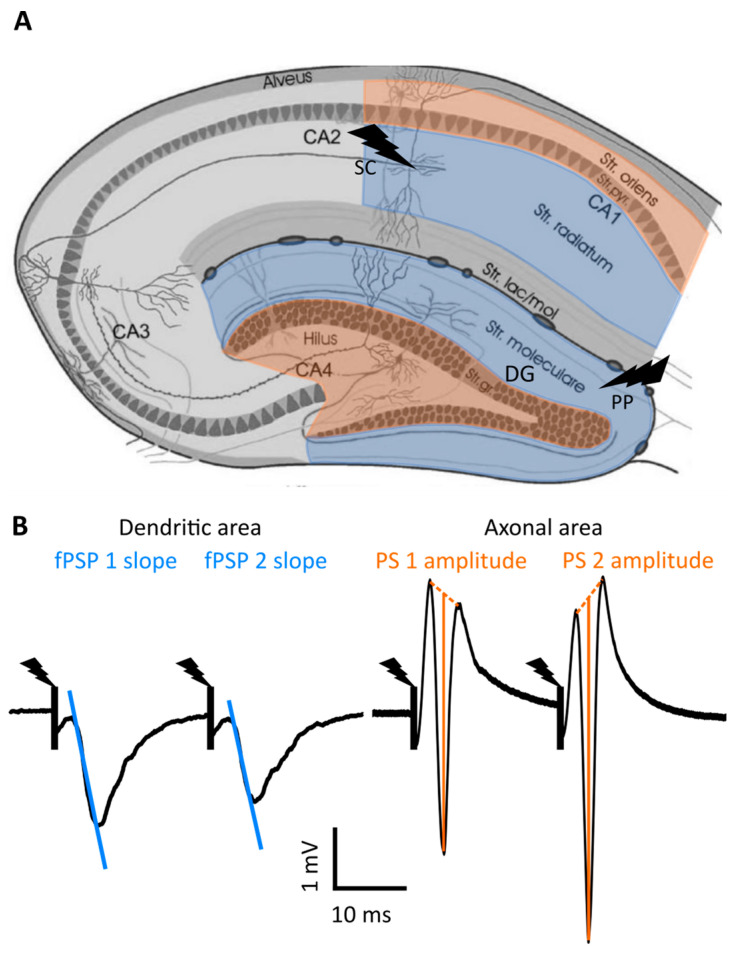
Schematic overview of the hippocampus with its electrophysiological responses. (**A**) Overview of the hippocampus with its regions and layers. Dendritic areas are depicted in blue (in which electrical stimulation can be applied, depicted by a lightning symbol), while axonal regions are shown in orange. (**B**) Example of negative and positive field postsynaptic potentials (fPSPs) measured in the dendritic or axonal areas, respectively, along with their outcome parameters. The stimulation artefact is marked with a black line and lightning symbol. DG, dentate gyrus; PP, perforant path; SC, Schaffer collaterals; PS, population spike. Figure adapted from [25].

Perforant path (PP) fibers form synapses with granule cells from the DG in the stratum moleculare (SM), whereas Schaffer collaterals (SC) form synapses with the pyramidal cells from the CA1 in the stratum radiatum (SR). Upon the stimulation of these fibers, excitatory neurotransmission takes place with inward currents of cations (sodium ions) in the dendrites of granule cells and pyramidal neurons, causing an active current sink in these dendritic areas and a passive current source in the cell bodies/axonal regions. Because these neurons are aligned along the same axis, a large current dipole is generated with a negative field potential in the dendritic regions and a positive field potential in the cell body/axonal regions. So, these negative fPSPs can be measured in the dendritic regions, i.e., the stratum radiatum (CA1) and the stratum moleculare (DG), and corresponding positive fPSPs in the axonal region, i.e., the stratum oriens (CA1) and the hilus (DG) (Figure 1). At higher stimulation intensities, associated with high levels of excitatory neurotransmission, large numbers of CA1 and DG neurons will generate action potentials within milliseconds, causing an active sink and negative potential, called a population spike (PS) in the cell body/axonal layers. A corresponding superposition of a positive potential could be measured in the dendritic region [22,23]. Based on the shape of the fPSP, inferences can be made about the excitability of the neurons. The initial slope of the negative fPSP is a measure for the strength of synaptic connectivity (the input), whereas the PS amplitude is a measure for neuronal output. Furthermore, after the administration of two electrical pulses spaced by tens of milliseconds, the ratio of the second to the first response can be calculated to investigate the short-term plasticity of the synaptic strength in local neuronal networks. A paired-pulse ratio higher or smaller than 1 is called paired-pulse facilitation or paired-pulse depression, respectively [21].

Numerous studies regarding fPSP recordings to evaluate hippocampal excitability have already presented distinct results that depend on the animal model and recording techniques [24,26,27]. An optimal method for these recordings, not yet used in this animal model, involves the use of a multielectrode array (MEA), as this device allows for the simultaneous monitoring of electrical responses from multiple locations in the same slice, providing valuable temporal and spatial information [28,29]. Moreover, the use of brain slices from animals or patients with mTLE should reflect the molecular and structural changes underlying epilepsy and better predict the efficacy of investigational therapies for the treatment of mTLE compared to healthy tissue. Ex vivo tests exploiting the kainic acid rat model have already been implemented in the recently modified epilepsy therapy screening program of the University of Utah [4,30,31].

For this study, we hypothesized that the morphological changes in the DG and CA1 subregion of the hippocampus in the IHKA mouse model are associated with modified circuit excitability, as measured through fPSP recordings in hippocampal slices.

## 2. Results

### 2.1. Input–Output Curve Analysis

fPSPs were recorded in hippocampal slices from healthy and IHKA mice with a 60 channel MEA after the electrical stimulation of the PP and SC for the DG and CA1, respectively. Input–output curves were obtained via the repeated administration of paired-pulses of increasing intensity (−500 to −3500 mV in steps of −250 mV). We selected one channel with the largest negative fPSP measured in the dendritic areas (negative fPSP hereafter referred to as fPSP) and compared the absolute value of the slope of the first and second fPSP as a measure for synaptic strength and the ratio of the slope of the second to the first fPSP as a measure for short-term plasticity (CA1: *n* = 31 (IHKA) and *n* = 22 (control); DG: *n* = 44 (IHKA) and *n* = 32 (control)). To explore differences in excitability, we selected one channel with the largest positive fPSP measured in axonal areas and compared the prevalence and amplitude of PS1 and PS2 (hereafter referred to as PS) (CA1: *n* = 31 (IHKA) and *n* = 25 (control); DG: *n* = 46 (IHKA) and *n* = 33 (control); see Table S1 for information about all animals and slices used in this study).

#### 2.1.1. fPSP Slope

**Figure 2:**
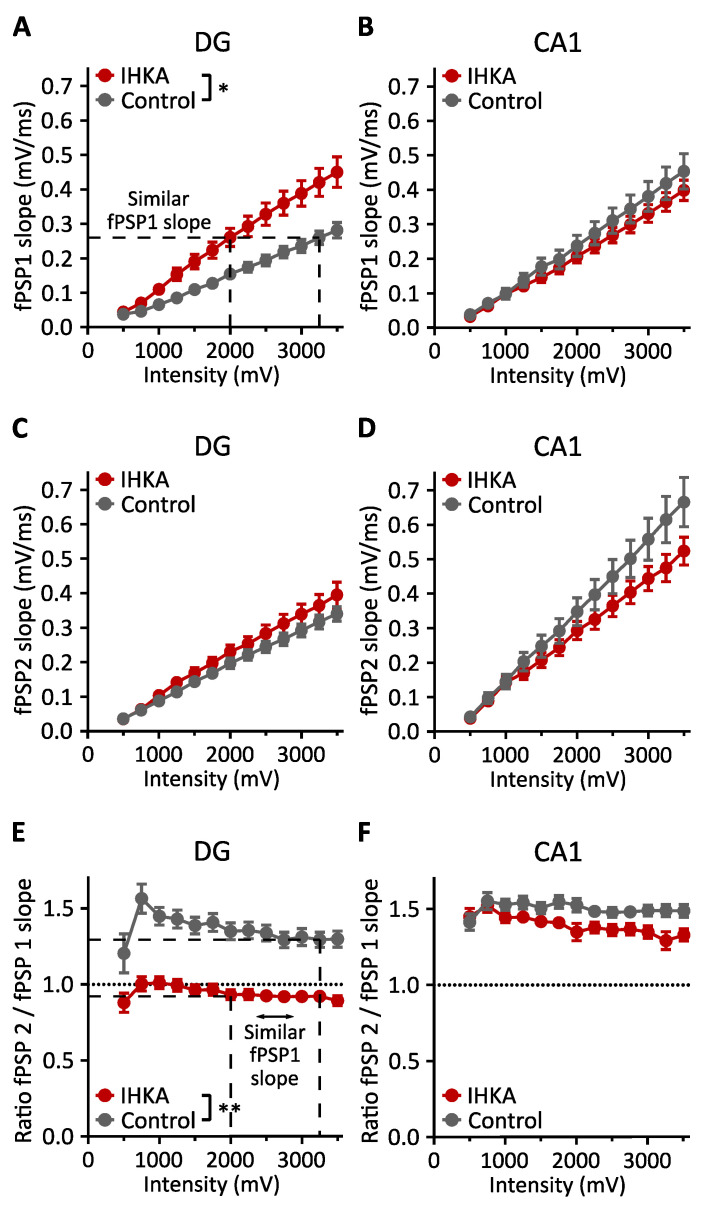
Differences in fPSP slope between the IHKA and control animals. (**A**,**B**) Input–output curves of fPSP1 slope measured in the DG and CA1 region, respectively. For the DG, a dashed line indicates a similar mean fPSP1 slope at different stimulation intensity values for the intrahippocampal kainic acid (IHKA) versus the control group. (**C**,**D**) Input–output curves of fPSP2 slope measured in the DG and CA1 region, respectively. (**E**,**F**) Input–output curves of the ratio of fPSP2 slope to fPSP1 slope for the DG and CA1 region, respectively. For the DG, a dashed line indicates the fPSP2/fPSP1 slope ratio for the IHKA and control group with a similar mean fPSP1 slope. Data are shown as mean ± standard error of the mean (SEM). * *p* < 0.05; ** *p* < 0.01.

In the DG, the increase in fPSP1 slope with increasing stimulation intensity was significantly larger in the IHKA (+0.038 mV/ms per 250 mV) versus the control group (+0.023 mV/ms per 250 mV, *p* < 0.05 for group-by-intensity interaction, linear mixed model). The mean fPSP1 slope that was reached at a stimulation intensity of 2000 mV for the IHKA group was only reached at a stimulation intensity of 3250 mV for the control group (Figure 2A). There was no significant difference between the IHKA and control animals for the fPSP2 slope (*p* > 0.5 for group effect, linear mixed model, Figure 2C). The ratio of the fPSP2 to fPSP1 slope was also significantly different between both groups (*p* < 0.01 for group-by-intensity interaction, linear mixed model, Figure 2E). In the IHKA group, the paired-pulse index was close to 1, with a trend to paired-pulse depression at high stimulation intensities, whereas in the control group, there was paired-pulse facilitation (which decreased with higher stimulation intensities). When comparing the ratio of the fPSP2 to fPSP1 slope at the stimulation intensities where the slices in the IHKA group exhibit a similar fPSP1 slope (I = 2000 mV) to that of the control group (I = 3250 mV), the same finding could be observed, i.e., that there is a significant difference between both groups (*p* < 0.001 for group effect, linear mixed model, Figure 2E).

In the CA1, there was no significant difference between the IHKA and control animals for the fPSP1 or fPSP2 slope (*p* > 0.5 for group effect, linear mixed model, Figure 2B,D) and for the ratio of the fPSP2 to fPSP1 slope (*p* > 0.05 for group effect, linear mixed model). For both conditions, paired-pulse facilitation was observed (Figure 2F).

#### 2.1.2. Population Spike

**Figure 3:**
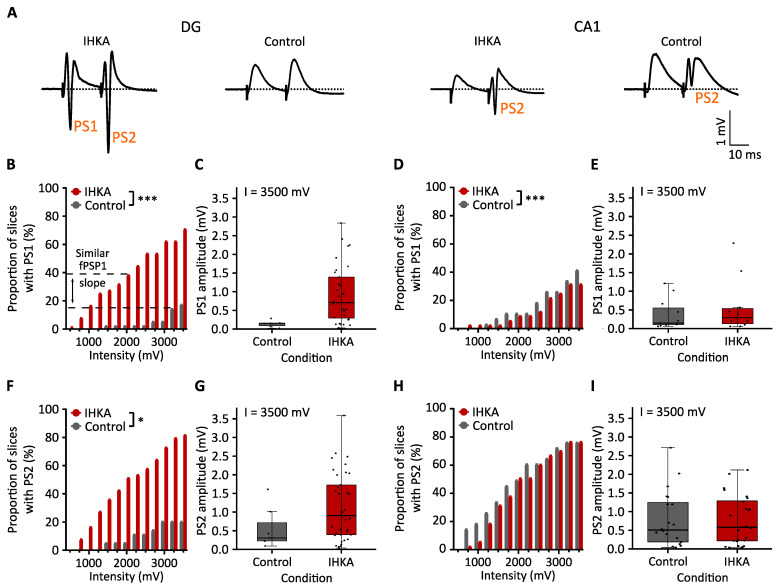
Differences in population spike between the IHKA and control animals. (**A**) Representative examples of fPSPs in IHKA and control slices measured in the DG and CA1 region, respectively, after maximal stimulation (3500 mV). The stimulation artefact is marked with a vertical black line. (**B**,**C**,**F**,**G**) Input–output curves of the proportion of slices with a PS and amplitude of the PS after the first and second stimulation, respectively, in the DG region. A dashed line indicates the proportion of slices with a PS1 at stimulation intensity 2000 mV for the IHKA group and 3250 mV for the control group (the stimulation intensity at which both groups display a similar mean fPSP1 slope). (**D***,***E**,**H**,**I**) Input–output curves of the proportion of slices with a PS and amplitude of the PS after the first and second stimulation, respectively, in the CA1 region. The proportion of slices data are shown as the effective proportion over all slices in our sample group for all stimulation intensities; the PS amplitude data are shown as mean ± SEM only for a stimulation intensity of 3500 mV. * *p* < 0.05; *** *p* < 0.001.

For the DG, the increase in the proportion of slices showing a PS1 and PS2 with increasing intensities was significantly higher in the IHKA group compared to the control group (*p* < 0.001 and *p* < 0.05 for group-by-intensity interaction, generalized estimating equation, GEE, logistic regression, Figure 3B,F). If the proportion of slices showing a PS1 at the stimulation intensities where the slices in the IHKA group exhibit a similar fPSP1 slope (I = 2000 mV) to that of the control group (I = 3250 mV) are compared, a trend towards a larger proportion of slices with a PS1 in the IHKA group is observed (*p* = 0.071 for group effect, GEE logistic regression). Due to the high variability in PS amplitude and the small subset of slices showing a PS in the control group, a statistical comparison of PS amplitude between the IHKA and control group did not reach significance. However, the graphs show a trend towards an increased PS1 and PS2 amplitude in the IHKA group compared to the control group for a stimulation intensity of 3500 mV (*p* > 0.05 for group effect, linear mixed model, Figure 3C,G).

The fPSP slope ratio, presented in Figure 2E, shows that the fPSP2 slope is similar or less steep than the fPSP1 slope in the IHKA group, while the fPSP2 slope is steeper than the fPSP1 slope in the control group (for all intensities but 500 mV) (based on the 95% confidence intervals, linear mixed model, see Supplementary Table S2). Based on this outcome, the occurrence and amplitude of PS2 and PS1 for increasing stimulation intensities were compared separately in the IHKA and the control group. In the IHKA group, the increase in the proportion of slices showing a PS2 versus a PS1 was not significantly different (*p* > 0.05 for ‘PS number’-by-intensity interaction, GEE logistic regression), while the increase in PS2 amplitude (+0.109 mV per 250 mV) was significantly larger in comparison to the PS1 amplitude (+0.078 mV per 250 mV, *p* < 0.001 for ‘PS number’-by-intensity interaction, linear mixed model). In the control group, the increase in the proportion of slices showing a PS2 was significantly larger than that for PS1 (*p* < 0.001 for ‘PS number’-by-intensity interaction, GEE logistic regression). The PS1 and PS2 amplitude were not compared due to the limited number of slices with a PS.

For the CA1, the increase in the proportion of slices with a PS1 with increasing stimulation intensity was significantly lower in the IHKA group compared to the control group (*p* < 0.001 for group-by-intensity interaction, GEE logistic regression, Figure 3D). There was no significant difference between both groups in the occurrence of PS2 (*p* > 0.05 for group effect, GEE logistic regression, Figure 3H). The PS1 amplitude and PS2 amplitudes were not significantly different between the groups (*p* > 0.05 for group effect, linear mixed model, Figure 3E,I). Across the groups, the increase in the proportion of slices showing a PS2 with increasing intensity was trend-significantly higher than for PS1 (*p* = 0.06 for ‘PS number’-by-intensity interaction, GEE logistic regression), but the increase in PS2 amplitude was not significantly higher than the increase in PS1 amplitude with increasing intensity (*p* > 0.05 for ‘PS number’-by-intensity interaction, linear mixed model). An overview of all the results of the input–output analysis is given in Table 1.

### 2.2. Spatial Analysis

**Figure 4:**
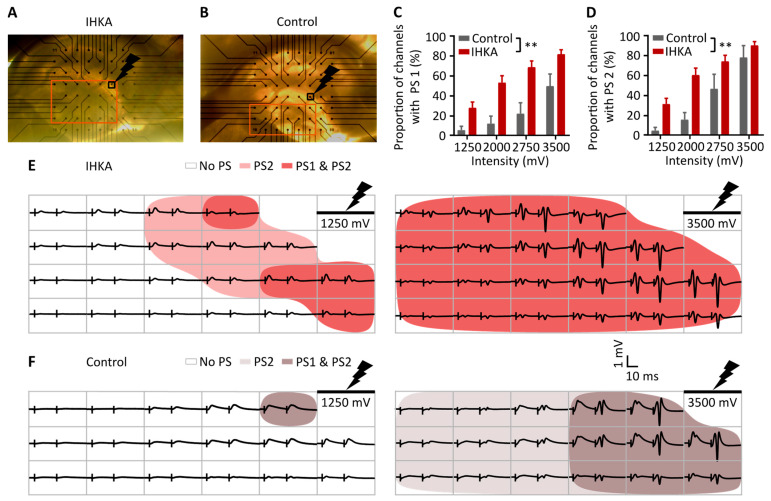
IHKA animals show a more widespread occurrence of population spikes. (**A**,**B**). Representative slices e of the IHKA and control group, respectively, with the channels in the hilus depicted in orange and the stimulation channel depicted in black with a lightning symbol. (**C**,**D**). Input–output curves of the proportion of channels with a PS1 and 2, respectively. Data are shown as mean ± SEM; ** *p* < 0.01. (**E**,**F**). Representative examples of fPSPs in the hilus in an IHKA and control slice after stimulation with a pulse of 1250 and 3500 mV. The color gradients indicate the occurrence of PS1 and/or PS2. The black vertical lines represent the stimulation artefacts.

Apart from the input–output analysis of a single channel, in MEA recordings, spatial information can be obtained as multiple channels are recorded simultaneously. To confirm the differences seen in the DG on a single channel level, the fraction of electrodes in the hilar region with a positive fPSP which also shows a superimposed PS was determined (*n* = 32 (IHKA) and *n* = 6 (control)). The minimum detection level for the positive fPSP and PS was set to 50 µV and 30 µV, respectively. The fraction of electrodes with a PS1 and PS2 was higher in the IHKA group compared to the control group for four stimulation intensities (1250, 2000, 2750, and 3500 mV), indicating more widespread activation (*p* < 0.01 for group effect, GEE logistic regression, Figure 4C,D).

## 3. Discussion

This ex vivo electrophysiology study, through comparing acute hippocampal slices from mice that were injected intrahippocampally with kainic acid or saline, mainly showed differences in the DG hippocampal subregion. Compared to the saline-injected mice, the slices of the IHKA mice showed (1) a steeper increase in fPSP1 slope with increasing stimulation intensities, which indicates an increased synaptic strength; (2) a paired-pulse fPSP slope ratio that is lower and close to 1, pointing towards a loss of paired-pulse facilitation of neurotransmission; and (3) an increased appearance and spatial extent of PS, revealing an increased granule cell output.

A possible reason for the increased synaptic strength is mossy fiber sprouting. These sprouted granule cell axons or mossy fibers make new synapses with other granule cells in the DG stratum moleculare. This phenomenon has been repeatedly observed in rodent models, including IHKA mice, as well as patients with mTLE [2,3,8,9,10,11,32,33,34,35]. This means that when electrical stimulation is applied to the molecular layer of the DG, it not only stimulates glutamatergic neurotransmission through the perforant path terminals but also activates it through the sprouted mossy fibers. Additionally, the increased synaptic strength can be attributed to dentate granule cell neurogenesis induced by seizures, which provides more dendrites from granule cells that are available for activation [36]. In the pilocarpine mouse model of mTLE, Hendricks et al. (2019) performed whole-cell voltage clamp recordings from hippocampal slices and showed that the optogenetic activation of neonatal- and adult-born granule cells often evoked excitatory postsynaptic currents (EPSCs) in opsin-negative DG granule cells, while this was rarely observed in slices from healthy control animals, confirming that sprouted mossy fibers in epileptic mice indeed form functional recurrent synapses. These sprouted mossy fiber synapses did show a strong paired-pulse synaptic depression. If indeed part of the stimulated synapses in DG of IHKA slices shows paired-pulse depression, this could explain the lack of paired-pulse facilitation [37]. Dysfunctional GABAergic inhibition could also partially explain our findings. Both in rodent models, especially the IHKA mouse model, and patients with mTLE, GABA_A_-mediated neuronal inhibition is profoundly compromised in the sclerotic hippocampus due to the loss of GABAergic interneurons and dysfunctional GABA_A_ receptors [12,38,39]. Sun et al. (2014) showed a reduced number of interneurons as well as a reduced GABA release in a rat model for temporal lobe epilepsy [40]. If feedforward inhibition resulting from the electrical activation of inhibitory synapses on granule cells is reduced, summated synaptic input would contribute to the steeper fPSP slopes in IHKA mice.

Apart from the increased synaptic strength, we observed a significantly higher granule cell output, reflected by a higher occurrence of PS1 and PS2 in the IHKA group. The previously mentioned increase in glutamatergic drive to the granule cells, as well as the decreased GABAergic inhibition, may also explain the increased postsynaptic activation upon stimulating the presynaptic components. Altered intrinsic excitability could also contribute to this finding as adult-born granule cells are hyperexcitable compared to neonatally born granule cells in IHKA animals [41,42]. This is further supported by the trend-significantly increased granule cell output (PS) for a similar synaptic input (fPSP slope) and by the spatially expanded granule cell output in IHKA versus control mice. Interpreting the latter should be carried out carefully, keeping in mind the complexity of spatial analysis [23].

There were almost no differences in the CA1 fPSPs between the hippocampal slices from the IHKA and healthy mice, suggesting that the overall synaptic strength and postsynaptic excitability of CA1 neurons in the intermediate hippocampus are barely affected. This was rather unexpected since pathophysiological changes expected to result in an increase in the synaptic strength and excitability of CA1 neurons have been described in the IHKA mouse model. A study by Malenka et al. (1991) demonstrated an increase in glutamatergic (AMPA) receptor expression in CA3 and CA1 neurons in rat hippocampal slices after treatment with picrotoxin [43]. Le Duigou et al. (2008) found that CA1 neurons have a depolarized resting membrane potential and GABA reversal potential and showed that interictal epileptiform discharges are generated in the CA1 and subicular regions in hippocampal slices from IHKA mice [13]. Also in hippocampal slices from IHKA mice, Kang et al. (2021) showed a loss of GABAergic interneurons without major changes in their intrinsic and synaptic properties (measured using the patch clamp method) [18]. These latter changes were, however, mainly found in the ventral ipsilateral or dorsal contralateral hippocampus and not in the region close to the kainic acid lesion, the site where we recorded from. In future studies, we should also record from ventral and/or contralateral hippocampal slices to test whether CA1 fPSPs are differentially affected in these regions. In the ipsilateral intermediate hippocampus, near the KA-injected dorsal hippocampus, reduced numbers of CA1/CA3 neurons are expected due to the excitotoxic effects of the kainic acid diffusing in this region [13,17]. Since we are recording activity from large groups of neurons, it is possible that increases in synaptic strength and/or neuronal excitability on a single neuron level are masked on a group level if the total number of neurons that is activated is reduced. To unmask such an effect, we could combine electrophysiology with the optical imaging of genetically encoded calcium/voltage sensors, as this would allow us to (semi-)quantify the number of activated neurons underlying the electrophysiological response to stimulation [44,45]. Moreover, in a future study, the link with the frequency of spontaneous seizures and their severity, as well as the time interval between SE and timepoint of decapitation, could be evaluated. In this study, EEG recordings were not performed to avoid the confounding effects of tissue damage induced by electrode implantation.

In the case of the intraperitoneal kainic acid rat model, the use of hippocampal slices has already been validated as a more cost-effective, less labor-intensive, and quicker testing option compared to in vivo studies for screening new therapies. The IHKA mouse model is becoming more popular due to its lower mortality rate, robust focal seizures, milder generalized seizures, and unilateral hippocampal sclerosis with granule cell dispersion [1,4]. Since mouse brains are smaller, ex vivo brain slices preserve more functional connectivity. The use of a MEA device allows for the simultaneous monitoring of electrical responses from multiple locations in the same slice, providing valuable temporal and spatial information. However, a compromise needs to be made between spatial coverage and resolution. In our study, we chose to have maximal coverage of hippocampal CA1 or DG, respectively, which came at the cost of having less spatial resolution. Therefore, it was not feasible to reproducibly select subregions of CA1 or DG for stimulation/recording, such as the lateral vs. medial perforant path or CA1a vs. CA1b vs. CA1c [28,29]. Another limitation of this approach is that not all circuits are preserved during the slicing process (e.g., the propagation of fPSPs evoked in the DG to the CA1 region, which was not feasible in our study design). This could be evaluated in a future study during in vivo fPSP recordings [29].

To conclude, we confirmed a disrupted dentate gate in the IHKA mouse model, characterized by increased excitatory neurotransmission and a greater activation of dentate granule cells in response to input stimuli. This ex vivo brain slice model has the potential to serve as a screening tool for restoring dentate gate function and developing new therapies for mTLE.

## 4. Materials and Methods

### 4.1. Animals

Fifty-five C57Bl/6J mice (seven weeks of age) were purchased from Envigo (the Netherlands). Only male mice were included to limit variability, as the reproductive cycle of females influences seizure frequency, and this was not assessed prior to the MEA experiments [46]. Animals were housed at a controlled temperature of 20–22 °C and at a relative humidity of 40–60%, with a fixed 12 h light/dark cycle and food and water provided ad libitum. Animals were group housed prior to and individually after intrahippocampal injection. All experiments were conducted in accordance with the relevant European guidelines (directive 2010/63/EU). The experiments were approved by the Animal Experimental Ethical Committee of Ghent University (ECD19/65).

### 4.2. Intrahippocampal Kainic Acid Injection and Status Epilepticus

SE was induced in eight-week-old mice (*n* = 36) via unilateral intrahippocampal injections of KA (200 ng kainic acid in 50 nL saline) in the dorsal right hippocampus, which resulted in electrophysiologically identifiable seizures, loss of CA1/CA3 pyramidal neurons, and granule cell dispersion in dorsal and intermediate hippocampal regions in our previous studies [47,48]. The control mice (*n* = 19) were intrahippocampally injected with vehicle solution (50 nL of saline). All animals were anesthetized with isoflurane (induction 5%, maintenance 2%) and medical O_2_ (induction 1 L/min, maintenance 0.8 L/min). They were secured in a stereotactic frame (Neurostar Robot Stereotaxic system, Neurostar, Tubingen, Germany) using ear bars. After exposure of the skull, a burr hole was made centered 2.0 mm posterior and 1.5 mm right to bregma. A blunt tip needle (25 gauge Hamilton, needle ref. 7001, Hamilton Company, Reno, NV, USA) connected to a 1 µl Hamilton syringe (syringe ref. 65458, Hamilton Company, Reno, NV, USA) was placed in the dorsal hippocampus according to the following stereotactic coordinates: [AP] −2.0 mm relative to bregma; [ML] +1.5 mm relative to bregma; [DV] −1.8 mm relative to brain surface. KA was injected at a rate of 100 nL/min using an automatic injector (StereoDrive, Neurostar, Tubingen, Germany). Five minutes after injection, the needle was slowly retracted, the skin was sutured, and the animals recovered from anesthesia. In the first few hours after the kainic acid injections, frequent behavioral arrest and sporadic clonic seizures were observed, indicating the occurrence of SE. Overall, 6 out of 36 KA injected mice died during SE, reducing the population to an IHKA group of 30 animals and a control group of 19 animals. At 4 to 7 weeks (80% of animals at 4–5 weeks, 10% of animals at 6 weeks, and 10% of animals at 7 weeks) after the intrahippocampal injection of KA or vehicle solution, the mice were anesthetized with 5% isoflurane, followed by decapitation for slice preparation.

### 4.3. Cortico-Hippocampal Slice Preparation and Positioning

Upon decapitation, the brain was quickly removed and submerged in ice-cold aCSF composed of (in mM) 124 NaCl, 2 KCl, 1.25 KH_2_PO, 2 CaCl_2_, 2 MgSO_4_, 26 NaHCO_3_, and 10 glucose. The aCSF was continuously carbonated (95% O_2_/5% CO_2_) to maintain a stable pH of 7.4. Horizontal cortico-hippocampal slices (350 µm) were prepared using a Leica VT1200S vibratome (Leica Microsystems, Wetzlar, Germany) and subsequently placed in carbogen-saturated aCSF at room temperature for at least one hour until use. Only slices from the ipsilateral intermediate hippocampus ([DV] 2–3.75 mm relative to brain surface) were selected, excluding the injected dorsal hippocampus, which demonstrates massive neuronal loss in the CA1, CA3, and hilar regions of the hippocampus [13,17].

For electrophysiological recordings, the slices were transferred to the MEA chamber (MultiChannel Systems (MCS), Reutlingen, Germany) and continuously superfused with carbogen-saturated aCSF at a flow rate of 2 mL/min. Slice positioning aimed for a maximum number of electrode contacts to be located at the level of the CA1 or DG region depending on the respective area under investigation (only one region was targeted per slice). Carbogen-saturated aCSF was preheated to 34 °C using a heatable perfusion cannula (PH01, MCS, Reutlingen, Germany) at the inlet of the MEA chamber where the temperature was maintained at 31 °C. Slice position was maintained by using a custom-made nylon grid. At least 15 min of equilibration time was allowed before starting the experiment.

### 4.4. Electrophysiological Set-Up

Field potentials were recorded with a planar 60 channel MEA (MCS, Reutlingen, Germany) containing 60 TiN-coated electrodes (30 µm diameter and 200 µm spacing). One of these electrodes was connected to the system’s ground and used as a reference electrode. Voltage signals were acquired using an MEA1060-BC high-bandwidth pre-amplifier and digitized at 25 kHz with 16-bit resolution using MC_Rack software (Version 4.6.2, MCS, Reutlingen, Germany). The stimulation parameters (duration, amplitude, and repetition rate) were controlled by a programmable stimulator (STG4002; MCS, Reutlingen, Germany).

### 4.5. CA1 and DG Evoked Potential Recording

After the equilibration period, a monophasic negative voltage pulse (−2000 mV, duration 100 µs) was applied to all of the electrodes located in the CA1 stratum radiatum containing the SC or in the DG stratum moleculare containing the PP (one by one) to identify the optimal stimulation site and to evaluate the slice quality. The optimal stimulation site was chosen to be the stimulated channel which resulted in the largest and clearest responses. Only experiments in which the fPSP recorded in at least four channels located in the CA1 stratum radiatum or in the DG stratum moleculare was greater than 250 µV and free from stimulation artifacts were included (Table S1). In all subsequent experiments, input–output curves were obtained via the repeated administration of paired monophasic negative pulses (20 ms interstimulus interval) of increasing intensity (−500 to −3500 mV in steps of −250 mV, 10 s interstep time). This protocol was repeated three times.

### 4.6. Data Analysis

**Figure 5:**
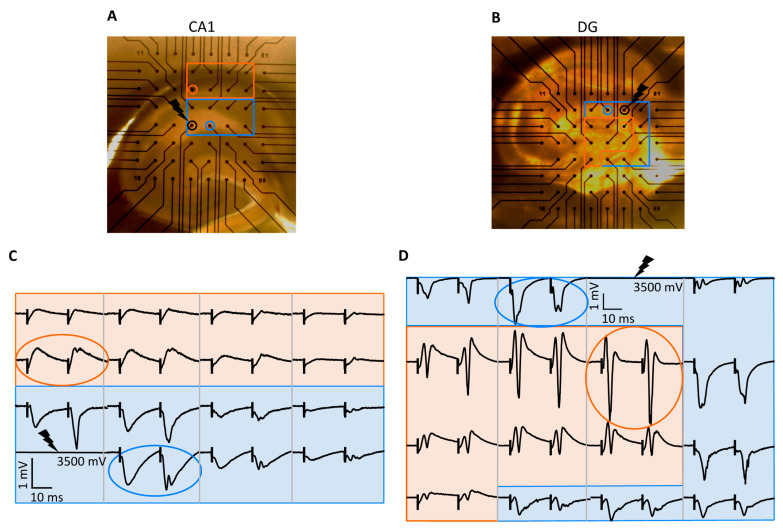
Examples of electrophysiological recordings. (**A**,**B**) Example of a hippocampal slice positioned on a MEA for the CA1 and DG recordings, respectively. The blue rectangle marks the dendritic area (i.e., the stratum radiatum and the stratum moleculare of the CA1 and DG, respectively). The orange rectangle marks the axonal area (i.e., the stratum oriens and the hilus of the CA1 and DG, respectively). (**C**,**D**) Examples of fPSPs measured in the CA1 and DG regions, respectively. The blue and orange background mark the negative and positive fPSPs, respectively. The black lightning symbol marks the stimulation electrode. The black vertical lines mark the stimulation artefacts. The chosen channels with the largest negative and positive responses of both regions are highlighted with a blue and orange circle, respectively, on both figures.

The offline analysis of fPSPs was performed using custom-written MATLAB software (version R2021b, MathWorks, Natick, MA, USA) and Microsoft Excel (version 2302, Microsoft 365, Redmond, WA, USA). For the input–output analysis, we selected two channels per slice: (1) the channel showing the largest negative fPSP without a corresponding positive PS (to obtain a ‘pure’ slope) in the stratum radiatum (CA1) or stratum moleculare (DG) and (2) the channel showing a positive fPSP with the largest PS in the stratum oriens (CA1) or hilus (DG) (Figure 5). The fPSP slope was calculated by fitting a tangent to the falling phase of the negative response using the least squares method. The PS amplitude was calculated as the vertical distance between the negative peak of the PS and the straight line connecting the positive peaks before and after the PS. These parameters were calculated for both responses (fPSP1 and fPSP2) of the paired-pulse stimulation. Once these parameters were obtained, the ratio of the fPSP2 and fPSP1 slope was calculated. From the three repeated measurements, the average of the parameters per intensity was calculated and used for further statistical analysis.

As differences in fPSP slope and PS occurrence/amplitude were observed in the DG between both groups, a spatial analysis was performed. Slices showing at least one channel with a PS larger than 30 µV were selected for this analysis. From these slices, we counted the total number of channels in the DG hilus which displayed an fPSP > 50 µV and the number of channels in which the fPSP displayed a PS > 30 µV for the following stimulation intensities: 1250 mV, 2000 mV, 2750 mV, and 3500 mV. The results are reported as proportion of channels with PS1 or PS2 relative to the number of channels showing a positive fPSP1 or 2 in the hilus.

### 4.7. Statistical Analyses

Statistical analyses were performed using IBM SPSS statistics for Windows (version 29.0, IBM corp., Armonk, NY, USA). As multilevel data (multiple slices clustered per animal) were used, differences in the continuous dependent variables (fPSP1 slope, fPSP2 slope, paired-pulse ratio, PS1 amplitude, and PS2 amplitude) explained by two independent variables (group and stimulation intensity) were analyzed by a linear mixed model analysis. The models included group (IHKA versus control), intensity, and group-by-intensity interaction as fixed effects and slices clustered in animals as random effects (as slices from the same animal are more likely to be similar than slices from different animals). In case of the fPSP1 and fPSP2 slopes, a linear regression was fit because a linear trend was observed in the relation between slope and stimulation intensity (for the intensity range applied in this study). We chose to use a random intercept and random slope linear model as this allowed the baseline fPSP (random intercept) and intensity dependency (random slope) to vary between slices. This approach corrects for the variability induced by differences in, e.g., slice positioning and slice health status. The AIC (Akaike Information Criterion) is used to select the best fitting model, i.e., the lower the AIC, the better the predicted values from the model fit the actual data [49,50].

Differences in dichotomous outcomes (i.e., the presence of PS1 and PS2) in the IHKA versus the control animals for different stimulation intensities were evaluated using a GEE logistic regression analysis with group (IHKA versus control), intensity, and group-by-intensity interaction as fixed effects. For the presence of PS1 and PS2 in the input–output analysis, the data of all slices were aggregated to have animals (and not slices) as subjects and intensity as the within-subject factor. The total number of slices from one animal was incorporated as the number of trials, whereas the number of slices with a PS1 or PS2 from one animal was incorporated as the number of events. For the proportion of channels with PS1 and PS2 in the spatial analysis, the model included slices clustered in animals as subjects and intensity as the within-subject factor. The total number of channels with a positive fPSP was incorporated as the number of trials, whereas the number of channels with a PS was incorporated as the number of events [50].

A two-tailed *p*-value of <0.05 was considered to be significant. All values are reported as mean ± standard error of the mean (SEM) unless stated otherwise, with *n* representing the number of slices. Data is visualized using the software Inkscape (version 0.92.4, www.inkscape.org).

## Figures and Tables

**Table 1:** Overview of differences in excitability between the IHKA and control animals.

Excitability Measure	Group	DG		CA1	
		Value *	Difference	Value *	Difference
**Synaptic strength**					
fPSP1 slope	IHKA	0.45 mV/ms	↑ *	0.40 mV/ms	ns
	Control	0.28 mV/ms		0.45 mV/ms	
fPSP2 slope	IHKA	0.40 mV/ms	ns	0.52 mV/ms	ns
	Control	0.34 mV/ms		0.67 mV/ms	
Paired-pulse index	IHKA	0.89	↓ **	1.49	ns
	Control	1.30		1.33	
**Neuronal output**					
Prevalence of PS1	IHKA	71.74%	↑ ***	32.26%	↓ ***
	Control	18.18%		42.31%	
Amplitude of PS1	IHKA	0.92 mV	ns	0.59 mV	ns
	Control	0.14 mV		0.38 mV	
Prevalence of PS2	IHKA	82.61%	↑ *	77.42%	ns
	Control	21.21%		76.92%	
Amplitude of PS2	IHKA	1.09 mV	ns	0.77 mV	ns
	Control	0.56 mV		0.79 mV	

* Values represented as the mean after electrical stimulation with maximal intensity (3500 mV). ns = no significant difference; ↑, increase; ↓, decrease in parameter in IHKA group compared to control group; * *p* < 0.05, ** *p* < 0.01, *** *p* < 0.001.

## Data Availability

The data presented in this study are available upon request.

## Supplementary Materials

The following supporting information can be downloaded at: https://www.mdpi.com/article/10.3390/ijms25010660/s1.
