# Supplementary material for: Increased Dentate Gyrus Excitability in the Intrahippocampal Kainic Acid Mouse Model for Temporal Lobe Epilepsy
Source: Int J Mol Sci. 2024 Jan 4;25(1):660. doi: 10.3390/ijms25010660 (PMC10779277; doi:10.3390/ijms25010660)
Supplement: Supplementary file 1 [file ijms-25-00660-s001.zip › ijms-2773782-supplementary.pdf]

Table S1: overview of all animals and slices used in this study.

**Group**	**Region**					
	CA1			DG		
	Animal	Number of slices	Time after injection	Animal	Number of slices	Time after injection
**IHKA**	11020	3	4 weeks	20322	2	6 weeks
	21020	1	4 weeks	30322	3	4 weeks
	40322	1	4 weeks	40322	1	4 weeks
	190321	3	5 weeks	70421	2	5 weeks
	191120	4	5 weeks	80621	4	5 weeks
	230321	2	4 weeks	90421	4	5 weeks
	241120	3	5 weeks	100621	2	5 weeks
	250321	4	4 weeks	160322	2	5 weeks
	251120	3	5 weeks	200322	4	4 weeks
	260321	2	4 weeks	210421	2	7 weeks
	261120	1	6 weeks	220322	4	4 weeks
	280920	1	4 weeks	230322	4	4 weeks
	300920	1	4 weeks	240322	2	4 weeks
	310820	1	7 weeks	280222	1	6 weeks
	27082020	1	7 weeks	280421	5	4 weeks
				290421	4	4 weeks
**Control**	10920	1	7 weeks	10621	4	4 weeks
	20621	1	4 weeks	20621	1	4 weeks
	30620	1	7 weeks	70621	4	5 weeks
	40620	2	7 weeks	80421	4	5 weeks
	171120	4	4 weeks	110621	2	5 weeks
	180321	5	5 weeks	170322	4	4 weeks
	181120	4	4 weeks	200421	4	7 weeks
	240321	3	4 weeks	270421	6	4 weeks
	300620	1	7 weeks	310322	4	4 weeks
	120221	3	/			
	150221	1	/			

Table S2: 95 % confidence intervals for the ratio of fEPSP2 to fEPSP1 slope in the DG (p<0.001 for group effect, linear mixed model,**).**

**Condition**	**Stimulation Intensity**	**95% Confidence Interval**	
	(mV)	Lower Bound	Upper Bound
**Control**	500	0.989	1.327
	750	1.325	1.608
	1000	1.351	1.664
	1250	1.303	1.547
	1500	1.267	1.499
	1750	1.290	1.517
	2000	1.232	1.458
	2250	1.239	1.463
	2500	1.223	1.445
	2750	1.176	1.399
	3000	1.187	1.412
	3250	1.177	1.401
	3500	1.177	1.411
**IHKA**	500	0.817	1.080
	750	0.896	1.120
	1000	0.866	1.122
	1250	0.875	1.073
	1500	0.865	1.052
	1750	0.871	1.053
	2000	0.828	1.009
	2250	0.840	1.019
	2500	0.832	1.009
	2750	0.826	1.004
	3000	0.826	1.006
	3250	0.828	1.007
	3500	0.795	0.983
